# Integron-mediated Multidrug Resistance in a Global Collection of Nontyphoidal *Salmonella* *enterica* Isolates

**DOI:** 10.3201/eid1503.081131

**Published:** 2009-03

**Authors:** Mary G. Krauland, Jane W. Marsh, David L. Paterson, Lee H. Harrison

**Affiliations:** University of Pittsburgh School of Medicine and Graduate School of Public Health, Pittsburgh, Pennsylvania, USA (M.G. Krauland, J.W. March, D.L. Paterson, L.H. Harrison); University of Queensland, Royal Brisbane and Women’s Hospital, Brisbane, Queensland, Australia (D.L. Paterson)

**Keywords:** Salmonella enterica, drug resistance, multidrug resistance, integrons, horizontal gene transfer, research

## Abstract

*Salmonella* *enterica* bacteria have become increasingly resistant to antimicrobial agents, partly as a result of genes carried on integrons. Clonal expansion and horizontal gene transfer may contribute to the spread of antimicrobial drug–resistance integrons in these organisms. We investigated this resistance and integron carriage among 90 isolates with the ACSSuT phenotype (resistance to ampicillin, chloramphenicol, streptomycin, sulfamethoxazole, and tetracycline) in a global collection of *S. enterica* isolates. Four integrons, *dfrA12/orfF/aadA2*, *dfrA1/aadA1*, *dfrA7*, and *arr2/blaOXA30/cmlA5/aadA2*, were found in genetically unrelated isolates from 8 countries on 4 continents, which supports a role for horizontal gene transfer in the global dissemination of *S. enterica* multidrug resistance. Serovar Typhimurium isolates containing identical integrons with the gene cassettes *blaPSE1* and *aadA2* were found in 4 countries on 3 continents, which supports the role of clonal expansion. This study demonstrates that clonal expansion and horizontal gene transfer contribute to the global dissemination of antimicrobial drug resistance in *S. enterica*.

## Abstract 2

Horizontal gene transfer and clonal expansion contribute substantially to the dissemination of resistant strains

*Salmonella enterica* bacteria are a leading cause of foodborne disease worldwide (*1**,**2*). In the United States, as many as 1.4 million cases of *S. enterica*–associated disease occur annually (*3*,*4*). While usually self-limiting, salmonellosis may require antimicrobial drug treatment in infants, the elderly, or immunocompromised persons. However, antimicrobial drug resistance has become increasingly common in *S. enterica*, which can complicate therapy. The National Antimicrobial Resistance Monitoring System reported that in 2004, 15.0% of non-Typhi isolates were resistant to >2 classes of antimicrobial drugs, and 8.1% were resistant to >5 classes. The most common *S. enterica* multidrug-resistance pattern in 2004 was ACSSuT (resistance to ampicillin, chloramphenicol, streptomycin, sulfamethoxazole, and tetracycline) (*5*).

Antimicrobial drug resistance can occur by point mutations in the bacterial genome or through horizontal transfer of genetic elements carrying resistance genes. Resistance may be disseminated through clonal expansion of drug-resistant strains or through horizontal transfer of genetic elements coding for resistance determinants. *S. enterica* populations change through the introduction of strains that expand and displace existing populations (*6*,*7*). Such population dynamics enable antimicrobial drug resistance in *S. enterica* to spread as a result of clonal expansion. The global dissemination of the multidrug-resistant (MDR) *S. enterica* serovar Typhimurim phage type DT104 clone is an example of the role of clonal expansion in the spread of antimicrobial drug resistance determinants across multiple countries and continents (*8*). Clonal expansion is also probably responsible for the dissemination of nalidixic acid resistance in *S. enterica* serovar Typhimurium isolates obtained in southern Asia and Africa (*9*).

Horizontal transfer of genetic material among *S. enterica* or from other bacterial species also plays an important role in the dissemination of drug resistance in this pathogen (*10*). Evidence indicates that horizontal gene transfer plays a major role in the dissemination of antimicrobial drug resistance in other bacterial species, such as *Escherichia coli* (*11*) and *Stenotrophomonas maltophilia* (*12*). The location of antimicrobial drug–resistant genes on mobile genetic elements, such as plasmids, transposons, and integrons, facilitates the mobilization of resistance from one organism to another (*13*).

Integrons are genetic structures capable of capturing and excising gene cassettes, which usually encode antimicrobial drug resistance determinants. Although integrons are not self-mobilizable, they are usually found in association with transposons and are often located on plasmids, facilitating their mobility (*13*). Integrons are thus ideally suited for the dissemination and recombination of antimicrobial drug–resistance genes. Integrons are common in *S. enterica* and make an important contribution to the extent of antimicrobial resistance in this species (*10*,*13*,*14*). Because of their plasmid and transposon association, integrons are assumed to be mobilized predominantly through horizontal gene transfer (*10*). However, the clonal nature of *S. enterica* suggests that clonal expansion may also play a role in dissemination of drug resistance. An example of clonal expansion of integron bearing *S. enterica* is the global distribution of the serovar Typhimurium DT104 clone, which harbors a genetic resistance island known as the *Salmonella* genomic island 1 (SGI1). This region contains a number of drug resistance elements including 2 integrons with the gene cassettes *blaPSE1* and *aadA2* and genes for tetracycline and chloramphenicol resistance, which are not integron associated (*15*).

Clonal expansion of integron-bearing *S. enterica* would account for the occurrence of a particular genetic lineage with a specific integron in a variety of regions. Horizontal gene transfer would account for the existence of identical integrons in isolates of different genetic lineages. To explore the roles of clonal expansion and horizontal gene transfer in the dissemination of antimicrobial drug resistance caused by class 1 integrons, we investigated the integron structure and genetic lineage of 90 MDR nontyphoidal *S. enterica* isolates from a global collection comprising >1,900 isolates from 13 countries and 6 continents. A goal of this study was to improve our understanding of the contributions of clonal expansion and horizontal gene transfer to the dissemination of integrons carrying antimicrobial drug–resistance genes in *S. enterica* to enable the development of improved strategies for the control of antimicrobial drug resistance in this organism as well as other emerging pathogens of public health importance.

## Materials and Methods

### Bacterial Isolates

A total of 1,920 *S. enterica* isolates were investigated; 1,743 isolates were collected by laboratories in Argentina, Australia, Belgium, Canada, Denmark, Germany, Italy, the Philippines, South Africa, Spain, Taiwan, Uganda and the United States during September 2001–August 2002 (Table 1). These isolates were collected as part of a separate study that attempted to identify a genetically and geographically diverse group of *S. enterica* isolates with reduced susceptibilities to fluoroquinolones. The isolates were not selected but rather were collected consecutively, without regard to their antimicrobial drug susceptibility. In addition, 179 isolates were collected by the Allegheny County Department of Health in Pennsylvania during 2002–2003 as part of routine surveillance. Serotyping was performed by the collecting laboratories, except for isolates from Taiwan, which were serotyped by the Pennsylvania Department of Health.

**Table 1:** Laboratories that provided *Salmonella enterica* isolates for this study

Country	Institution	Contact person
United States	Centers for Disease Control and Prevention Foodborne and Diarrheal Diseases Laboratory Section, Atlanta	Timothy J. Barrett
Canada	Ontario Public Health Laboratory, Toronto	Frances Jamieson
Canada	Laboratory for Foodborne Zoonoses, Population and Public Health Branch, Guelph	Cornelius Poppe
Argentina	Centro de Estudios en Antimicrobianos, Buenos Aires	Jose Maria Casellas
Australia	Queensland Health Scientific Services, Archerfield	John Bates
Belgium	Antwerp University Hospital, Antwerp	Herman Goossens
Germany	Bundesgesundheitsministerium für gesundheitlichen Verbraucherschutz und Veterinärmedizin, Berlin	Andreas Schroeter
South Africa	South African Institute for Medical Research, Johannesburg	Karen Keddy
Spain	Institute of Health Carlos III, Enteric Bacteria Laboratory, Madrid	Miguel Usera
Italy	Istituto Superiore di Sanita, Rome	Alessandra Carattoli
Denmark	Hvidovre Hospital, Copenhagen	Dennis Hansen
Taiwan	National Cheng Kung University, Tainan City	Wen-Chien Ko

The ACSSuT resistance phenotype has become increasingly prevalent in *S. enterica*, and that phenotype has been commonly associated with class 1 integron carriage in this species. For these reasons, we selected a subset of isolates from the collection that exhibited the ACSSuT resistance phenotype for further investigation. Isolates selected for integron investigation were confirmed to be *S. enterica* by PCR with primers specific for the *invA* region of the *inv* locus (*16*) (Table 2).

**Table 2:** Primers used for PCR amplification of *Salmonella enterica* integrons

Primer	Sequence (5′ → 3′)	Target	Reference
5’CS	GGCATCCAAGCAGCAAGC	5′ conserved segment	(*17*)
3’CS	AAGCAGACTTGACCTGAT	3′ conserved segment	(*17*)
int_F	CGATGCGTGGAGACCGAAACCTT	intI1	(*18*)
int_R	GTAACGCGCTTGCTGCTTGGATGC	intI1	(*18*)
invA_F	ACACAGCTCGTTTACGACCTGAAT	invA	(*16*)
invA_R	AGACGACTGGTACTGATCGATATT	invA	(*16*)
sul1_F	GCGCGGCGTGGGCTACC	sul1	This study
sul1_R	CCGCAAGGCTCGCTGGAC	sul1	This study
aadA1_R	CGATGACGCCAACTACCTCTGATA	aadA1internal primer	This study
arr2_F	ATTGTTGGCGTTGTTGAAGACTGG	arr2 internal primer	This study
cmlA5_F	GAATGGGAATGGGATGCCTGATAG	cmlA5 internal primer	This study
oxa10_R	TTTACAAAGCACGAAGACACCATT	blaOXA10 internal primer	This study
cmlA_F	GCAGGTCGCGAGGAAAGTAATG	cmlA 5′ forward primer	This study
cmlA_R	ACACCGCCCAAGCAGAAGTAGA	cmlA 3′ reverse primer	This study
blaOXA30_F	TCGCAAGAAATAACCCAAAAA	blaOXA30 internal primer	This study
aacA4_F	AAGCGGGGTTTGAGAGG	aacA4 forward primer	This study
aacA4_R	CGCGTACTCCTGGATCGGTTTCTT	aacA4 reverse primer	This study
dfr_1_F	TTTAGGCCAGTTTTTACCCAAGAC	dfrA1 internal primer	This study
ere_est_R	GCGCCAGCAGAATTATCCTTACAT	ereA2 internal primer	This study
aac(6’)IIC_F	CCGCGGGATTGACCAGT	aac(6′)IIC internal primer	This study
dfrA12_F	GCTGCGCATTTTGGTTCC	dfrA12 internal primer	This study
aadA2_R	TGTCATTGCGCTGCCATTCTCC	aadA2 internal primer	This study
qacH_F	GCGTCGCCGTTCTAAATCTGCTAT	qacH internal primer	This study
aac_R	GGGCGCCGGGTGTCTGGAG	aacA4 internal primer	This study
IS_F	GTCACGCCCCGACCATCACCTTCC	IS1247 internal primer	This study
TNP_F	CCGCGCTGGCCGACCTGAAC	Transposase A internal primer	This study
ere_F	CCTAACCGGGCGATTCAA	Erythromycin esterase internal primer	This study
cmlA_R_internal	ATCACACGCCCCATAAAACGAG	cmlA internal primer	This study
arr_R2	GCGGGATCCAGAACCAGGCGACAT	arr-2 internal primer	This study
arr_accA_R	AGAGCGGCTTTGGTTCC	Internal primer arr-2–accA junction	This study
ere_F2	CGCTGATTTCGCTGTCCTGA	ereA internal primer	This study
dfrA17_F	AAAAAGGCTAACAAGTCGT	dfrA17 internal primer	This study
cml_R2	GCTGAATTGTGCTCGCTGTCGTA	cml internal primer	This study
aadA_con_F	CGACATCATYCCGTGGCGTTAT	aadA forward consensus primer	This study
aadA_con_R	CGGCAGCCACATCCTTC	aadA reverse consensus primer	This study
aacA4_F	ATGACCTTGCGATGCTCT	aacA4 internal primer	This study
aacA4_R	CTCGATGGAAGGGTTAGG	aacA4 internal primer	This study
blaOXA30_F	ACACAATACATATCAACTTCGC	blaOXA30-aadA internal primer	This study
aadA1_R_S	GGATAACGCCACGGAATGATGTC	aadA1 internal primer	This study
albany_PSE1a_F	CCTTTGGGGCCACCTACAG	blaPSE1 primer	This study
albany_PSE1b_F	ATCAAAATTATGGGGTTACTTACA	blaPSE1 primer	This study
albany_dfr1_F	ATGGTAGCTATATCGAAGAATGGA	dfr primer	This study
albany_dfr2_F	AAGTACTGGCTATTGCCTTAGGAG	dfr primer	This study
U7-L12	ACACCTTGAGCAGGGCAAAG	SGI1 left junction	(*15*)
LJ-R1	AGTTCTAAAGGTTCGTAGTCG	SGI1 left junction	(*15*)
104-RJ	TGACGAGCTGAAGCGAATTG	SGI1 right junction	(*15*)
104-D	ACCAGGGCAAAACTACACAG	SGI1 right junction	(*15*)

### Antimicrobial Drug Resistance Testing

Antimicrobial drug resistance was determined by using the disc diffusion method on Mueller–Hinton agar (Becton, Dickinson and Co., Sparks, MD, USA), according to the manufacturer’s directions. Susceptibility to ampicillin, chloramphenicol, streptomycin, sulfamethoxazole, and tetracycline was determined according to the manufacturer’s breakpoints.

### Integron Detection and Characterization

Genomic DNA from *S. enterica* isolates was prepared using the DNeasy Tissue Kit (QIAGEN, Valencia, CA, USA), according to the manufacturer’s directions. Class 1 integron carriage was determined by PCR using primers specific to the *intI* region of the integrase gene. Isolates positive for integrase were further characterized by PCR using primers specific for the 5′ and 3′ conserved segments (CS) of the integron structure. PCR was performed in a 50-µL volume consisting of 1× PCR buffer II (Applied Biosystems, Foster City, CA, USA), 1.5 mmol/L MgCl_2_, 0.1 mmol/L dNTPs, 0.33 µM forward and reverse primers, and 1.66 units Amplitaq Gold polymerase (Applied Biosystems). PCR conditions were an initial denaturation at 95°C for 5 min; 35 cycles at 95°C for 1 min, 58°C for 1 min, and 72°C for 5 min plus 5 s each cycle (5 s longer in each subsequent cycle than in the previous cycle); and a final extension at 72°C for 7 min.

Long-range PCR was performed to detect integrons >2.0 kb by using the Gene Amp HiFidelity Kit (Applied Biosystems), according to the manufacturer’s directions. Long-range PCR conditions were an initial denaturation at 94°C for 2 min; 10 cycles at 94°C for 15 s, 58°C for 30 s, and 68°C for 4 min, followed by 20 cycles at 94°C for 15 s, 58°C for 30 s, and 68°C for 4 min plus 5 s each cycle (5 s longer in each subsequent cycle than in the previous cycle); and a final extension at 72°C for 7 min. PCR products were subjected to electrophoresis on 1% agarose gels, stained with ethidium bromide, and visualized using UV illumination on a Gel Doc 2000 documentation system (Bio-Rad, Hercules, CA, USA). For isolates that amplified multiple integrons, PCR products were separated by gel electrophoresis and purified by using either the Qiaquick Gel Extraction Kit (QIAGEN) or the Quantum Prep Freeze ’N Squeeze DNA Gel Extraction Spin Column Kit (Bio-Rad). Some PCR products were cloned before sequencing by using the Topo TA Cloning Kit for Sequencing (Invitrogen, Carlsbad, CA, USA) according to the manufacturer’s directions.

Isolates in this study were investigated for the presence of SGI1 and variant SGI1s by PCR using published primers specific for the right (104-RJ, 104-D) and left (U7-L12, LJ-R1) junctions of the chromosomal insertion site (*19*). Isolates were considered positive for the left or right junctions of the SGI1 if they generated PCR product of the appropriate size with primers specific for that junction. By this method, isolates with the SGI1 would be positive for the left junction but not for the right junction because of the presence of a retronphage between the 104-RJ and 104-D primer sites. Isolates with variant SGI1s would be positive for the left and right junctions.

### Gene Cassette Identification

Class 1 integron gene cassettes were identified by PCR and sequence analysis by using the 5′ and 3′ CS primers. Sequencing was performed with the BigDye terminator 3.1 kit (Applied Biosystems) according to the manufacturer’s instructions. Capillary sequence analysis was performed on a 3730 DNA sequence analyzer (Applied Biosystems). Sequences were analyzed and additional primers were designed by using the Lasergene 7.0.0 software package (DNAStar, Madison, WI, USA). Gene cassette homology searches were performed by using BLAST analysis (www.ncbi.nlm.nih.gov/BLAST) (*20*).

Ninety-one of the 121 integrons found in this study were sequenced in their entirety. In some cases, when integrons were identified that were of the same size as those previously sequenced, their gene cassettes were identified by PCR using primer pairs designed in this study (Table 2).

### Multilocus Sequence Typing

Genetic relatedness of isolates was assessed using multilocus sequence typing (MLST). MLST uses sequences ≈500 bp in length from 7 housekeeping genes to define a sequence type (ST). Isolates with the same alleles at all 7 loci are considered to be genetically indistinguishable by MLST and therefore define an ST. Isolates with the same alleles at 6 loci are considered to be closely related genetically.

MLST was performed using the 7-locus scheme described on the *Salmonella* MLST database (http://web.mpiib-berlin.mpg.de/mlst) (*21*–*24*). Visualization of PCR products and sequencing of gene fragments was accomplished as described above for integron gene cassettes. Sequences were analyzed using Bionumerics software V 5.10 (Applied Maths, Austin, TX, USA). Alleles and STs were assigned by the *Salmonella* MLST database. All isolates in this study and their associated sequence types have been deposited in the *Salmonella* MLST database.

### Definitions of Horizontal Gene Transfer and Clonal Expansion

Horizontal gene transfer was defined as *S. enterica* isolates belonging to different STs (STs that differ at >1 locus) but bearing the same integron. Clonal expansion was defined as *S. enterica* isolates bearing the same integron and belonging to the same ST or STs differing at only 1 locus but occurring in >1 location. Because isolates in this study were collected consecutively over a limited time in each location, and because source was the only epidemiologic information available, we could not determine whether genetically related isolates bearing the same integron in a given location were part of an outbreak or whether the isolates reflected clonal expansion beyond an outbreak. Therefore, *S. enterica* isolates collected from 1 location that belonged to the same ST and harbored identical integron structures were considered to be 1 isolate for classification as either horizontal gene transfer or clonal expansion.

## Results

### ACSSuT Resistance

Of the 1,920 isolates initially screened by antimicrobial drug susceptibility testing, 104 (4.9%) exhibited the ACSSuT resistance phenotype. The proportion of ACSSuT-resistant isolates ranged from 0% in Australia, Argentina, Belgium, and Canada to 19% in Taiwan and South Africa (Table 3).

**Table 3:** Source and ACSSuT resistance in a global collection of *Salmonella enterica* isolates*

Source	Total no. isolates	No. (%) ACSSuT-resistant isolates
Argentina	148	0
Australia	146	0
Belgium	66	0
Canada	144	0
Denmark	153	8 (5.2)
Germany	150	1 (0.7)
Italy	156	3 (1.9)
Philippines	67	6 (8.9)
Spain	151	8 (5.3)
South Africa	160	30 (18.8)
Taiwan	150	29 (19.3)
United States/ACHD	179	8 (4.5)
United States/CDC	150	1 (0.7)
Uganda	100	10 (10.0)
Total	1,920	104 (5.4)

*ACSSuT, ampicillin, chloramphenicol, streptomycin, sulfamethoxazole, and tetracycline; ACHD, Allegheny County Health Department; CDC, Centers for Disease Control and Prevention.

### Integron Detection and Characterization

Of the 104 nontyphoidal *S. enterica* isolates with the ACSSuT resistance phenotype, 90 (86.5%) were positive for the integrase gene and amplified gene cassette regions by PCR with primers for class 1 integron 5′ and 3′ CS regions. Most cassette-positive isolates contained either 1 (n = 61, 68%) or 2 (n = 26, 29%) integrons (Appendix Table). Three isolates contained >3 integrons. Although 16 different integrons were found in this collection, 19 distinct integron profiles could be identified because of multiple integrons in some isolates (Appendix Table). Six integrons contained only 1 gene cassette: *aacA4*, *aadA2*, *aadB*, *blaPSE1*, *dfrA7*, or *dfrA15*. Seven integrons contained 2 or 3 gene cassettes (listed in order of cassette occurrence in the individual integron): *aadB/catB3, aac3A-Id/aadA7*, *blaOXA30/aadA1*, *dfrA12/orfF/aadA2*, *dfrA1/orfC*, *dfrA1/aadA1*, and *tnpA/dfrA7.* A 4.0 kb integron containing the cassettes *arr2/cmlA5/blaOXA10/aadA1*was found alone or in combination with an integron containing the single gene cassette *aacA4*. Two other unique large integrons were found in this collection: a 5.8-kb integron with the gene cassettes *qacH/dfrA17/ereA/aadA2/cmlA/aadA1* and a 6.0-kb integron with cassettes *aac(6')-IIc/ereA2/IS1247/aac3/arr/ereA2*.

A total of 121 class 1 integrons were identified. Of these, 91 were fully sequenced (Appendix Table). With 2 exceptions, genetic drift in gene cassette sequences was not observed. One integron containing the single cassette *aadA2* showed 1 base difference from other *aadA2* gene cassettes found in this study (a T→C transition at position 39 of the gene cassette, resulting in a synonymous change). One *blaOXA30* gene cassette exhibited a point mutation (A→G) at position 31, also a synonymous change. All other gene cassettes within the study showed 100% nucleotide identity to GenBank reported cassettes, except for the *dfrA17* gene cassette identified in the unique 5.8-kb integron in the Stanley isolates from Taiwan (Appendix Table). This cassette showed 91% sequence identity to a gene cassette found in uncultured bacteria (GenBank accession no. FM179325) and in *E. coli* (GenBank accession no. EU687490).

Most gene cassettes found in this study confer resistance to the aminoglycosides (*aadA1*, *aadA2*, *aadA7*, *aadB*, *aacA4*, *aac*, *aac3A-Id*, *aac(6')-IIc*), trimethoprim (*dfrA1*, *dfrA7*, *dfrA12*, *dfrA15*, *dfrA17*), and β-lactams (*blaPSE1*, *blaOXA10*, *blaOXA30*). Other resistance cassettes included those coding for chloramphenicol resistance (*cmlA, cmlA5, catB3*), erythromycin resistance (*ereA2*), rifampin resistance (*arr2*), and resistance to quaternary ammonium compounds (*qacH*). Because resistance to the aminoglycoside streptomycin and the β-lactam ampicillin were selection criteria for isolates in this study, the predominance of cassettes encoding resistance to those antimicrobial drugs is not unexpected.

Phenotypic resistance to chloramphenicol was also a selection criterion, but only 3 integrons contained gene cassettes for this resistance. None of the integrons identified in this study carried genes coding for tetracycline resistance, although phenotypic resistance to this antimicrobial drug was also an inclusion criterion. Because the SGI1 contains genes for chloramphenicol and tetracycline resistance, which are not located within integrons, tetracycline- and chloramphenicol-resistant isolates that are SGI1 positive most likely contain these genes. Alternatively, resistance to chloramphenicol and tetracycline may be encoded elsewhere on the chromosome or on structures other than integrons in isolates that are not positive for the SGI1.

The SGI1 was identified in 17 isolates (19%) (Appendix Table). Twelve of the SGI1-positive isolates were serovar Typhimurium, belonged to ST 19, and showed the integron pattern (*blaPSE1*, 1.0 kb and *aadA2*, 1.2 kb) characteristic of serotype Typhimurium phage type DT 104. An additional Typhimurium ST 19 isolate containing 4 integrons, including *blaPSE1* and *aadA2*, was also positive for SGI1. Four serotype Albany isolates ST 292 were positive for a variant SGI1, which includes the integrons *dfrA1/orfC* and *blaPSE1* (*15*). This result indicates chromosomal location of these integrons in these isolates.

### Genetic Relatedness of Integron-bearing Nontyphoidal *S. enterica*

The 90 integron-containing *S. enterica* isolates represented 17 different STs. Thirty-three (37%) Typhimurium isolates belonged to ST 19 or to STs that differ from 19 at only 1 locus. These isolates contained 10 different class 1 integrons, which combined to create 12 integron profiles (Appendix Table). Eleven (12%) isolates of serovar Isangi belonged to ST 216 or to STs that differ from 216 at 1 or 2 loci. These isolates contained 3 integrons and 4 integron profiles. The remaining isolates represented diverse STs, which differed from each other at 6 or 7 of the MLST loci.

### Integron Distribution Across Nontyphoidal *S. enterica* Genetic Lineages

This study identified 5 class 1 integrons that were distributed across different genetic lineages, supporting the role of horizontal gene transfer in the dissemination of antimicrobial drug resistance in nontyphoidal *S. enterica*. The class 1 integron *dfrA12/orfF/aadA2*, which confers resistance to trimethoprim and streptomycin/spectinomycin, was identified in *S. enterica* from 5 different serotypes belonging to 5 STs (Figure, panel A). This integron was geographically widespread, being found in isolates from Europe, the United States, Taiwan, the Philippines, and South Africa. The integron containing the single trimethoprim-encoding *dfrA7* cassette was present in isolates from 2 serotypes, 2 STs and 3 different areas (Figure, panel B). The integron *dfrA1/aadA1* was found in isolates from 3 serotypes, 2 STs, and 2 areas (Figure, panel C).

**Figure:**
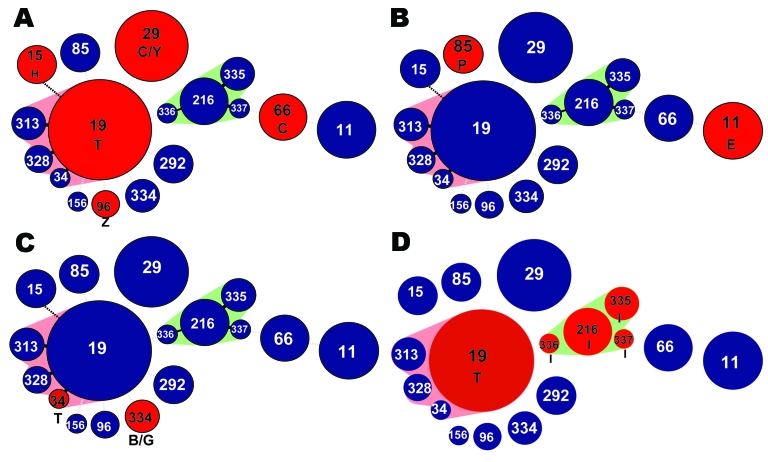
Minimum spanning trees depicting integron distribution across *Salmonella enterica* genetic lineages. A) dfrA12/orfF/aadA2; B) dfrA7; C) dfrA1/aadA1; D) arr2/blaOXA30/cmlA5/aadA2. Circles represent unique sequence types (STs). Red circles represent the STs that carried the integron involved in horizontal gene transfer. Numbers in circles represent the ST. Circle size reflects number of isolates in each ST. Pink and green shading indicates closely related groups of isolates. Letters refer to serotypes: B, Brandenburg; C, Cholerasuis; E, Enteriditis; H, Heidelberg; G, Goettingen; I, Isangi; P, Paratyphi A; Z, Schwarzengrund; Y, Stanley; T, Typhimurium. Geographic sources of isolates are as follows: Panel A: ST66, serotype C, Taiwan; ST29, serotype C, Taiwan; ST29, serotype Y, Taiwan; ST96, serotype Z, Denmark and Taiwan; ST19, serotype T, US Centers for Disease Control and Prevention and South Africa; ST15, serotype H, Philippines; Panel B: ST11, serotype E, Uganda and South Africa; ST85, serotype P, Denmark; Panel C: ST334, serotype G, Spain; ST334, serotype B, Spain; ST34, serotype T, Germany; Panel D: ST19, serotype T, South Africa; ST216, ST335, ST336, and ST 337, serotype I, South Africa.

Isolates of ST 216, 335, 336, and 337 (serovar Isangi) and ST 19 (serovar Typhimurium) from South Africa contained an identical 4.0 kb integron not previously reported in *S. enterica*, with the cassettes *arr2/cmlA5/blaOXA10/aadA1* (Figure, panel D). In some serovar Typhimurium ST 19 isolates, this integron was found in combination with the *aacA4* integron. The presence of a unique integron in genetically unrelated isolates from the same area indicates that this integron may have been horizontally transferred.

An integron containing the single gene cassette *aacA4* was found alone in isolates of serovar Isangi with ST 335 from Uganda and South Africa. This integron was also found in serovar Typhimurium isolates with ST 19, both alone and in combination with the integron *arr2/cmlA5/blaOXA10/aadA1*. These isolates belong to different genetic lineages, in that they differ at all 7 MLST loci. The occurrence of the same integron in these genetically unrelated isolates further supports a role for horizontal gene transfer in the dissemination of antimicrobial drug resistance in nontyphoidal *S. enterica*.

### Evidence for Clonal Expansion of Integron-mediated MDR in *S. enterica*

The serotype Typhimurium phage type DT104 integron pattern, with 2 class 1 integrons of sizes 1.0 and 1.2 kb and bearing the gene cassettes *blaPSE1* and *aadA2*, was found in 12 (13%) serovar Typhimurium isolates of ST 19 (Table 4) and in 1 additional serovar Typhimurium ST 19 isolate, which also contained 2 other integrons. This result is consistent with the hypothesis that a common ancestor has undergone clonal expansion in a number of areas.

**Table 4:** Evidence of clonal expansion among isolates from a global collection of *Salmonella enterica* isolates*

Integron profile	No. isolates	Serotype	Sequence type	Source
*blaPSE1*, *aadA2*	4	Typhimurium	19	United States/ACHD
	2	Typhimurium	19	Spain
	3	Typhimurium	19	Italy
	3	Typhimurium	19	South Africa
*blaOXA30/aadA1*	1	Typhimurium	328	Philippines
	2	Typhimurium	19, 328	Taiwan
	1	Typhimurium	313	South Africa
*aadB/catB3*	1	Typhimurium	19	Taiwan
	1	Typhimurium	328	Philippines
*dfrA12/orfF/aadA2*	1	Schwarzengrund	96	Taiwan
	1	Schwarzengrund	96	Denmark
*dfrA12/orfF/aadA2*	1	Typhimurium	19	United States/CDC
	2	Typhimurium	19	South Africa
*aacA4*	1	Isangi	335	South Africa
	1	Isangi	335	Uganda
*dfrA7*	6	Enteritidis	11	Uganda
	2	Enteritidis	11	South Africa

*ACHD, Allegheny County Health Department; CDC, Centers for Disease Control and Prevention.

Serovar Typhimurium isolates of ST 19 from Taiwan and ST 328 from the Philippines contained 2 class 1 integrons with the cassettes *aadB/catB3* and *blaOXA30/aadA1*. The integron *blaOXA30/aadA1* was also found alone in an isolate of serovar Typhimurium ST 328 from Taiwan and in combination with an integron containing the cassette *aadB* in an isolate of serovar Typhimurium ST 313 from South Africa. ST 313 and ST 328 are closely related to ST 19, differing from it at only 1 locus (ST 313 differs from ST 19 at the MLST locus sucA, ST 328 and ST 19 differ at MLST locus aroC; see Appendix Table). Therefore, these isolates are all closely related, and their integrons may represent an instance of clonal expansion.

Although the class 1 integron *dfrA12/orfF/aadA2* appears to have been circulated through horizontal gene transfer, this integron has also spread through clonal expansion of nontyphoidal *S. enterica*. This integron appeared in isolates of serovar Schwarzengrund ST 96 in Taiwan and Denmark and in serovar Typhimurium isolates of ST 19 in the United States and South Africa. Similarly, integrons with the single gene cassettes *aacA4* and *dfrA7* also exhibited both horizontal gene transfer and clonal expansion. The *aacA4* integron was found in serovar Isangi isolates of ST 335 in Uganda and South Africa. The *dfrA7* integron was found in serovar Enteritidis isolates of ST 11, also in Uganda and South Africa.

## Discussion

To better understand the dissemination of integron-mediated antimicrobial drug resistance, this study characterized the class 1 integrons and genetic lineages associated with 90 multidrug-resistant isolates obtained from a global collection of nontyphoidal *S. enterica*. Integrons found in this collection were diverse in size, gene cassette combination and distribution, and presented evidence for roles of clonal expansion and horizontal gene transfer.

Horizontal gene transfer is an important factor in the dissemination of antimicrobial drug–resistance genes, particularly when those genes are associated with mobile elements such as plasmids, transposons, and integrons (*10*,*11*,*14*). In this study, the widespread distribution of the *dfrA12/orfF/aadA2* integron among several STs and across several distinct regions is an example of horizontal gene transfer, as is the presence of the integrons *dfrA7*, *dfrA1/aadA1*, and *arr2/cmlA5/blaOXA10/aadA1* in different genetic backgrounds (when assessed by MLST). These integrons are potentially capable of transmitting drug resistance to other *S. enterica* isolates or to other bacteria.

Integrons are widely distributed among bacterial species. The integron found in the greatest number of different genetic lineages in this study, *dfrA12/orfF/aadA2*, has been previously reported in a number of species, including *E. coli* (GenBank accession no. AF335108, unpub. data), *Serratia marcesens* (Genbank accession no. AF284063, unpub. data), and *Salmonella* (*25*). The integron *dfrA1/aadA1* has been documented in *E. coli* from Turkey (*26*) and cited in *E. coli* in GenBank entries from India (GenBank accession no. EF417897, unpub. data) and Kenya (GenBank accession no. EF417897, unpub. data), in *Klebsiella pneumonia* from Poland (GenBank accession no. AY007807, unpub. data), and in *S. enterica* (GenBank accession no. AM746675, unpub. data). Transfer of integrons between different bacterial species has been documented in the clinical setting, which poses a serious threat to containment of nosocomial infections (*27*). The existence of identical integrons in different types of bacteria and the ability of these integrons to be transferred in vivo indicates that many bacteria acquire integrons from a common pool. This is an important consideration in efforts to halt the development and spread of antimicrobial drug resistance.

In this study, clonal expansion of *S. enterica* appears to be responsible for a large fraction of the dissemination of drug resistance integrons. Although several integrons demonstrated evidence of clonal expansion, including *aacA4*, *aadB/catB3*, *blaOXA30/aadA1*, *dfrA7*, and *dfrA12/orfF/aadA2,* the strongest evidence is presented by the prevalence and geographic ubiquity of the serotype Typhimurium DT104 pattern clone, in which the resistance-encoding integrons (*blaPSE1* and *aadA2*) are chromosomally integrated. These integrons are still mobilizable (*28*) but chromosomal location may make them more likely to be disseminated through clonal expansion than through horizontal gene transfer, particularly in the absence of antimicrobial selective pressure.

Our study assessed mechanisms of dissemination of integrons in a collection that is more genetically and geographically diverse than is typical for studies of integrons in *S. enterica*. MLST, used for assessment of genetic relatedness of isolates in this study, is better suited to analysis of global populations than other commonly-used methods, such as pulsed-field gel electrophoresis. In addition, this study focused on the relative contributions of clonal expansion and horizontal gene transfer to the dissemination of class 1 integron borne genes coding for antimicrobial drug resistance, which has not previously been well explored.

Antimicrobial drug resistance is a serious and increasing problem in *S. enterica* and in other gram-negative pathogens, such as *Pseudomonas aeruginosa, Acinetobacter baumannii*, *K. pneumoniae* and *E. coli* (*29*). The genes that code for antimicrobial drug resistance in these pathogens have proven to be remarkably mobile and widely distributed within and between species. The dissemination of integrons bearing antimicrobial drug resistance gene cassettes in *Salmonella* and other bacteria is a complex process that involves both the horizontal transfer of mobile genetic elements and the expansion of particularly fit clones. The combined effect of these mechanisms is that, if integrons confer an adaptive benefit caused by the presence of antimicrobial drug selective pressure or if clones harboring these integrons have increased fitness caused by other factors, then the integrons may disseminate rapidly both geographically and among diverse species. It is important to understand these mechanisms of transmission to develop methods for surveillance and control of antimicrobial drug resistance.

## Supplementary Material

Appendix Table Description of integrons and associated serotypes, MLST results, and countries of origin of 90 isolates with the ACSSuT phenotype used in this study*
