# Supplementary material for: Integron-mediated Multidrug Resistance in a Global Collection of Nontyphoidal Salmonella enterica Isolates
Source: Emerg Infect Dis. 2009 Mar;15(3):388–96. doi: 10.3201/eid1503.081131 (PMC2666292; doi:10.3201/eid1503.081131)
Supplement: Appendix Table — Description of integrons and associated serotypes, MLST results, and countries of origin of 90 isolates with the ACSSuT phenotype used in this study* [file 08-1131_appT-s1.pdf]

Appendix Table. Description of integrons and associated serotypes, MLST results, and countries of origin of 90 isolates with the ACSSuT phenotype used in this study*

Integron profile†	No. isolates (no. with integrons sequenced)	Serotype	MLST results							ST (no. isolates)	Country
			aroC	*dnaN*	*hemD*	*hisD*	*sucA*	*purE*	*thrA*		
aacA4	1 (1)	Isangi	117	73	41	79	71	65	72	335 (1)	South Africa
	1 (1)	Isangi	117	73	41	79	71	65	72	335 (1)	Uganda
	1 (1)	Typhimurium	10	7	12	9	5	9	2	19 (1)	South Africa
aac(6')-IIc/ereA2/IS1247/aac/arr/ereA2	3 (2)	Heidelberg	2	7	9	9	5	9	12	15 (3)	Philippines
aadA2	1 (1)	Isangi	2	73	41	79	71	65	72	216 (1)	South Africa
arr2/cmlA5/blaOXA10/aadA1	8 (5)	Isangi	2	73	41	79	71	65	72	216 (5)	South Africa
			117	73	41	79	71	65	72	335 (1)	
			10	73	41	79	71	9	72	336 (1)	
			2	73	41	79	71	65	8	337 (1)	
	3 (1)	Typhimurium	10	7	12	9	5	9	2	19 (3)	
blaOXA30/aadA1	1 (1)	Typhimurium	116	7	12	9	5	9	2	328 (1)	Taiwan
dfrA1/aadA1	1 (1)	Goettingen	11	10	13	32	10	13	112	334 (1)	Spain
	2 (2)	Brandenburg	11	10	13	32	10	13	112	334 (2)	
	1 (1)	Typhimurium	10	19	12	9	5	9	2	34 (1)	Germany
dfrA12/orfF/aadA2	5 (5)	Cholerasuis	34	31	35	14	26	6	8	66 (4)	Taiwan
			16	16	20	18	8	12	18	29 (1)	
	1 (1)	Stanley	16	16	20	18	8	12	18	29 (1)	
	1 (1)	Schwarzengrund	43	47	49	49	41	15	3	96 (1)	
	1 (1)	Schwarzengrund	43	47	49	49	41	15	3	96 (1)	Denmark
	1 (1)	Typhimurium	10	7	12	9	5	9	2	19 (1)	United States/CDC
	1 (1)	Heidelberg	2	7	9	9	5	9	12	15 (1)	Philippines
	2 (2)	Typhimurium	10	7	12	9	5	9	2	19 (2)	South Africa
dfrA7	6 (5)	Enteritidis	5	2	3	7	6	6	11	11 (6)	Uganda
	2 (2)	Enteritidis	5	2	3	7	6	6	11	11 (2)	South Africa
	4 (2)	Paratyphi A	45	4	8	44	27	9	8	85 (4)	Denmark
qacH/dfrA17/ereA/aadA2/cmlA/aadA1	13 (3)	Stanley	16	16	20	18	8	12	18	29 (13)	Taiwan
tnpA/dfrA7	1 (1)	Enteritidis	5	2	3	7	6	6	11	11 (1)	Uganda
aacA4, *arr2/cmlA5/blaOXA10/aadA1*	6 (3)‡	Typhimurium	10	7	12	9	5	9	2	19 (6)	South Africa
aadA2, *blaOXA30/aadA1*	1 (1)	Typhimurium	10	7	12	9	112	9	2	313 (1)	South Africa
aadB, *blaOXA30/aadA1*	1 (1)	Typhimurium	10	7	12	9	112	9	2	313 (1)	South Africa
aadB/catB3, *blaOXA30/aadA1*	1 (1)	Typhimurium	10	7	12	9	5	9	2	19 (1)	Taiwan
	1 (1)	Typhimurium	116	7	12	9	5	9	2	328 (1)	Philippines
blaPSE1, *aadA2*	4 (4)	Typhimurium§	10	7	12	9	5	9	2	19 (4)	United States/ACHD
	2 (2)	Typhimurium§	10	7	12	9	5	9	2	19 (2)	Spain
	3 (3)	Typhimurium§	10	7	12	9	5	9	2	19 (3)	Italy
	3 (3)	Typhimurium§	10	7	12	9	5	9	2	19 (3)	South Africa
dfrA1/orfC, *blaPSE1*	4 (4)	Albany§	104	100	54	78	104	9	48	292 (4)	Taiwan
dfrA15, *blaPSE1*, *aac3A-Id/aadA7*	1 (1)	Newport	63	14	6	12	5	14	58	156 (1)	Denmark
aadB, *aadA2*, *blaOXA30/aadA1*	1 (1)	Typhimurium	10	7	12	9	112	9	2	313 (1)	South Africa
dfrA15, *blaPSE1*, *aadA2*, *dfrA12/orfF/aadA2*	1 (1)	Typhimurium§	10	7	12	9	5	9	2	19 (1)	United States/ACHD

*ACSSuT, ampicillin, chloramphenicol, streptomycin, sulfamethoxazole, and tetracycline; MLST, multilocus sequence typing; ST, sequence type; CDC, Centers for Disease Control and Prevention; ACHD, Allegheny County Health Department.
†Order of gene cassettes is as listed.
‡3 aacA4 cassettes were fully sequenced; *arr2/cmlA5/blaOXA10/aadA1* gene cassettes were identified by partial sequencing and PCR with multiple primer pairs internal to gene cassettes.
§Positive for SGI1 or variant SGI1.
